# A Second Gastrotomy for the Removal of a Foreign Body from the Œsophagus

**Published:** 1888-01

**Authors:** 


### *A SECOND GASTROTOMY FOR THE REMOVAL OF A FOREIGN BODY FROM THE ŒSOPHAGUS.*

It was the privilege of the readers of the JOURNAL, last December, to read an account of an operation, at once original and successful, by Dr. M. H. Richardson, for the removal of a foreign body from the lower end of the œsophagus, where it had resisted attempts to dislodge it from above, and was too low to be reached by an œsophagotomy. Dr. Richardson opened the stomach by an incision parallel with the border of the ribs, inserted the hand and fore-arm into the stomach, introduced two fingers into the œsophagus, and drew the foreign body—a dental plate with false teeth—into, and then out of, the stomach.

A second gastrotomy for a foreign body, this time a peach-stone, has just been published by Dr. Wm. T. Bull, of New York. The subject of the operation was a colored boy, sixteen years of age, and the operation itself differed in certain features from the prior operation of Richardson. The incision was three inches in length, from the level of the ninth costal cartilage to two inches above the umbilicus; the upper limit was determined by noting the point where percussion dullness of the liver disappeared. This incision was chosen because an incision in the median line would be nearer the œsophageal opening than one parallel with the border of the ribs, and the hand or finger, operating through it, would be less encumbered by the overhanging ribs. This incision exposed a part of the anterior wall of the stomach and its greater curvature about two inches and a-half from the pylorus. At the part chosen for the incision into the stomach, two loops of silk were placed two inches apart in a vertical line, and the incision made an inch and a-quarter between them; additional loops in the edges gave greater control over the incision. The finger, inserted into the stomach through this incision, closed the wound entirely, and was passed directly backward until the vertebral column was felt, and then upward till it entered the œsophagus, thus making the vertebræ a guide to the œsophagus. The anterior wall of the stomach, with its loops of silk, followed the silk into the cavity, and was folded on itself like the invaginated scrotum in examination of the vaginal canal. The foreign body was felt, and gentle attempts were made to extract it through the stomach. These failing, a slender bougie was passed along the finger, up the

œsophagus, and projected from the mouth; a sponge was attached by a strong silk to the lower end, and pulled through with a view to drag the peach-stone up from below. The first attempt was unsuccessful, but a second, with a larger sponge, brought the stone into the mouth, when the finger met and extracted it. A rapid recovery, without accident, followed the operation.

The noticeable features of the case, which contributed to its success, are: The small wound in the stomach, just sufficient to admit the index finger, the invagination of the anterior wall of the stomach, the control of the stomach wound by loops of thread, so it was held closed against the finger, which acted as a plug and prevented the escape of the gastric fluids, and the very moderate manipulation of the stomach itself.

Dr. Bull emphasizes the advisability, when there is doubt as to the possibility of safely extracting a foreign body by œsophagotomy, of resorting promptly to opening the stomach.—*Boston Medical and Surgical Journal.*

